# Application of Linear Mixed-Effects Model, Principal Component Analysis, and Clustering to Direct Energy Deposition Fabricated Parts Using FEM Simulation Data

**DOI:** 10.3390/ma17205127

**Published:** 2024-10-21

**Authors:** Syamak Pazireh, Seyedeh Elnaz Mirazimzadeh, Jill Urbanic

**Affiliations:** Department of Mechanical, Automotive and Materials Engineering, University of Windsor, 401 Sunset Ave, Windsor, ON N9B 3P4, Canada; pazireh@uwindsor.ca (S.P.); mirazims@uwindsor.ca (S.E.M.)

**Keywords:** direct energy deposition, machine learning, principal component analysis, self-organizing maps, linear mixed-effects models

## Abstract

The purpose of this study is to investigate the effects of toolpath patterns, geometry types, and layering effects on the mechanical properties of parts manufactured by direct energy deposition (DED) additive manufacturing using data analysis and machine learning methods. A total of twelve case studies were conducted, involving four distinct geometries, each paired with three different toolpath patterns based on finite element method (FEM) simulations. These simulations focused on residual stresses, strains, and maximum principal stresses at various nodes. A comprehensive analysis was performed using a linear mixed-effects (LME) model, principal component analysis (PCA), and self-organizing map (SOM) clustering. The LME model quantified the contributions of geometry, toolpath, and layer number to mechanical properties, while PCA identified key variables with high variance. SOM clustering was used to classify the data, revealing patterns related to stress and strain distributions across different geometries and toolpaths. In conclusion, LME, PCA, and SOM offer valuable insights into the final mechanical properties of DED-fabricated parts.

## 1. Introduction

Direct energy deposition (DED) additive manufacturing (AM) is an advanced manufacturing process used for repairing damaged parts, adding features, and building new parts from 3D model data on a metal additive basis. This process involves joining materials in a stack-by-stack and layer-by-layer manner, with the initial material stock being either in powder or wire form [1]. During the additive process, material is added and energy is applied (laser, beam, arc) to form the melting zone. As the deposition follows the predefined toolpath, the already-built beads solidify as they cool down to lower temperatures, a process that can be tracked and observed using image-filtering tools [2]. The thermal diffusion within the part during the build and cooling-down processes has a significant impact on the intrinsic residual stresses, strains, and distortions of the material [3]. The thermal history and temperature gradients have a microscopic impact on the material’s strength through grain formation, which is affected by these temperature gradients [4]. As stresses and strains within fabricated parts vary significantly with temperature gradients, it is essential to pay attention to process parameters [5], geometry [6], and deposition toolpath patterns [7] that affect the temperature history of the built parts.

High fabrication costs, random errors, instability, and significant computational demands in DED necessitate leveraging big data from tests and simulations. Data analysis can help identify the root causes of part quality abnormalities in DED manufacturing, although correlation may be low within post-process data, necessitating in-situ tracing of fusion phenomena [8]. Machine learning has shown promising results in gaining stable fabrication in the laser-directed energy deposition [9]. Convolutional neural networks (CNNs) have demonstrated potential for developing a predictive model based on a large dataset of real-time image processing to control DED manufacturing [10]. Li et al. collected experimental data from a DED study to predict grain boundary tilt based on thermal gradients, crystal orientation, and the Marangoni effect using an artificial neural network (ANN) structure [11]. The analytical model obtained from a trained ANN provides a valid and fast prediction of grain growth behavior in DED parts.

Machine learning can also recognize patterns, correlations, and dependencies between geometrical and mechanical properties of DED-fabricated parts. A defect classification was proposed by Patil et al. for DED processes [12]. Xu et al. [13] proposed a hybrid approach combining deep learning and mixed-effect modeling, where the random effect accounts for mean temperature variations for real-time defect detection. Unsupervised clustering has also been used in the research [14] to categorize the geometries based on the accuracy of the manufacturing quality.

The toolpath and geometry types have shown an inter-coupled effect on the qualities of DED-fabricated parts [3]. A clustering approach used in [15] investigated stress-distortion feature-based analysis of multiple geometries to determine local point assignments to clusters. This approach provided insight into the physical similarities among edge and internal locations of the observations. However, the aforementioned clustering approach did not account for toolpath effects when generating the FEM-based simulation data.

In the current research, we conducted a statistical analysis across four different geometric shapes, each paired with three distinct toolpath patterns (resulting in twelve separate case studies). The goal was to investigate the impact of toolpath patterns, geometry types, and layering effects on mechanical properties, with a particular focus on residual stresses and strains. Analyses were performed on finite element (FEM) simulation data, which includes element birth and death in a multilayer, multi-track DED physics setting. All node data were extracted. Directional residual stresses, strains, and maximum principal stresses were chosen as the physical features of concern. A correlation analysis was conducted to determine how these features are related. The analysis was applied to the entire dataset as well as to datasets for each geometry and toolpath separately. Then, the linear mixed-effects model was investigated to consider the toolpath pattern, geometry type, and layering effect as fixed variables on the local nodes’ data (the observations), with the observations treated as random variables. The linear mixed-effects (LME) model determines the contribution of each fixed variable to the mechanical properties. A principal component analysis (PCA) determines what features have the highest variance and are more informative in DED-processed data. The results are followed by a self-organizing map (SOM) clustering approach to compare the thin and thick longitudinal and transverse wall data at different layers to see how the toolpath and geometry create similar properties within various parts and deposition patterns.

## 2. Materials and Methods

The case studies, material, and analysis methodology are described in this section.

### 2.1. Case Studies

**Figure 1:**
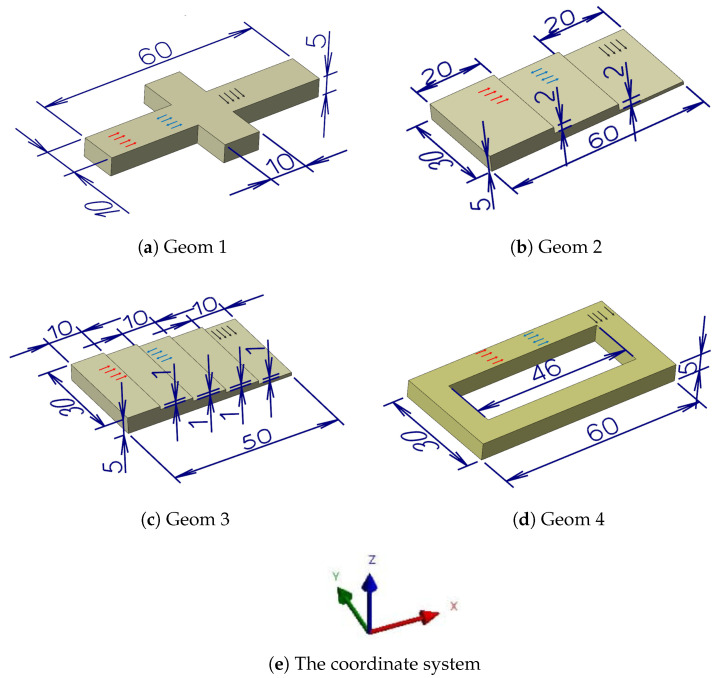
The geometries considered for this research include a cross-type, a 3-step plate, a 5-step plate, and a rectangle with a hole, with dimensions in millimeters. The red arrows represent the one-way longitudinal toolpath, the blue arrows represent the longitudinal zigzag toolpath, and the black arrows represent the one-way transverse zigzag for each geometry.

Four distinct geometries, each with three separate deposition toolpath patterns, are analyzed in this study. The geometries include a cross-type, a 3-step plate, a 5-step plate, and a rectangle with a hole. For each geometry, three toolpaths are examined: one-way longitudinal, longitudinal zigzag, and one-way transverse. The term “longitudinal” refers to the longitudinal direction of the parts. Figure 1 shows the geometries considered for the research. The schematic of the toolpath on each geometry is depicted with three colored arrows corresponding to the coordinate system. In total, 12 distinct case studies are considered. Hereafter, the x-axis refers to the longitudinal direction, and the y-axis refers to the transverse direction. The APlus add-on from CAD/CAM Mastercam software (Mastercam 2025, V27.0.6876.0) [16] was used to generate the laser metal AM toolpath NC files. All the case studies have the same substrate with a dimension of 90 (mm) × 60 (mm) × 7 (mm). The materials for this study are 316L stainless steel (SS) and A36 steel for the clad and the substrate, respectively.

The process parameters of the fabrications are presented in Table 1. In the present study, the term “overlapping” pertains to the region of interlocking between two beads (or two tracks).

The FEM simulation data with bilinear elastoplastic modeling were used as the basis for data analysis in this research. The element birth and death technique, utilizing ANSYS APDL (2023 R2) programming, was employed to activate deposition elements according to the toolpath pattern. A Python script was developed to read the NC files and mesh element data. Then, in the pre-processing step, the order of the activated elements and their corresponding IDs were determined. Once the elements were ordered, the APDL programming added each element individually to mimic the deposition physics. The mesh size for the DED process is determined by the beads’ width and depth, adopting a strategy where each element representing the melt zone is activated on an individual basis. The width of each element reflects the bead’s non-overlapped area, ensuring that the bead count across the depositions matches the number of deposition tracks extracted from the NC files from APlus software (an Add-Ons of Mastercam). Note that the validity of the FEM model was assessed through a comparison with experimental measurements and a study by the authors in [17]. The results of [17] show that the model accurately captures the stress gradients aligned with the actual physics at the corner sides of the model where heat diffusion is well represented. However, the middle of the model may exhibit an overprediction of residual stress due to an overestimation in the thermal model, yet the stress gradient pattern remains similar to the actual physics. There are limitations in simplifying the model as the material overlap cannot be addressed with the FEM simulations.

The simulations were conducted on the Digital Resource Alliance of Canada cloud accounts, each equipped with 128 GB RAM and 8 CPU cores. Processing and post-processing of all cases took several weeks. An ANSYS post-processing APDL script was developed to extract data for all nodes within the range of simulation sub-steps. The data for all the FEM nodes were stored in a dataset. The current study focused on statistical and machine learning analyses of the data to gain deeper insight into the physics by interpreting the data. These analyses were applied using Python scripts. In the following subsections, the statistical and machine learning methods used in the study are introduced.

### 2.2. Correlation Analysis

Correlation measures how closely variables or dataset features approximate linear functions. The relationship between two features will always be higher if it is closer to some linear function, so the linear correlation between them will be stronger, and the correlation coefficient will be greater in absolute value.

Considering a dataset with two features: $\vec{x}$ and $\vec{y}$, each with *n* number of observations, the “Pearson” correlation coefficient is measured by the following:

(1) $$r=\frac{\sum_{i=1}^{n}\left(x_{i}-\bar{x}\right)\left(y_{i}-\bar{y}\right)}{\sqrt{\sum_{i=1}^{n}{\left(x_{i}-\bar{x}\right)}^{2}\sum_{i=1}^{n}{\left(y_{i}-\bar{y}\right)}^{2}}}$$

where $\bar{x}$ and $\bar{y}$ are the mean values of the features:

(2) $$\bar{x}=\frac{\sum_{i=1}^{n}x_{i}}{n}$$

(3) $$\bar{y}=\frac{\sum_{i=1}^{n}y_{i}}{n}$$

The Pearson correlation values, *r* (Equation (1)), range between −1 and 1, and as the absolute value increases, the correlation between the two features rises.

### 2.3. Linear Mixed-Effects Model

LMEs are used for regression analyses involving dependent data when data are collected and summarized in groups. The statsmodels [18] implementation of LME is primarily group-based, meaning that random effects must be independently realized for responses (observations) in different groups.

Some specific linear mixed-effects models are as follows [18]:

- Random intercepts models: In these models, all responses within a group are additively shifted by a value that is specific to the group.
- Random slopes models: The responses in a group follow a conditional mean trajectory that is linear in the observed covariates, with both slopes (and possibly intercepts) varying by group.
- Variance components models: The levels of one or more categorical covariates are associated with draws from distributions. These random terms additively determine the conditional mean of each observation based on its covariate values.

The coefficients of these models may vary according to the grouping variables used to describe the relationship between the response variable and the independent variables. The mixed-effects model consists of two parts, fixed effects, and random effects. Generally, fixed-effects terms represent linear regression, while random effects represent randomly selected experimental units.

The random coefficients are defined as follows:

(4) $$Y_{ij}=\beta_{0}+\beta_{1}Z_{ij}+\gamma_{0i}+\gamma_{1i}Z_{ij}+\epsilon_{ij}$$

where $Y_{ij}$ is the *j*th measured response (observation) for subject (group) *i*, and $Z_{ij}$ is the covariate for this response. The “fixed effects parameters”, $\beta_{0}$ and $\beta_{1}$, are shared by all subjects, and the error term $\epsilon_{ij}$ is independent of the parameters and distributed with a mean of zero. The “random effects parameters”, $\gamma_{0i}$ and $\gamma_{1i}$, also follow a bivariate distribution with a mean of zero.

The statsmodelsMixedLM treats the entire dataset as a single group to include crossed random effects in a model. The variance components in the model are used to define models with various combinations of crossed and non-crossed random effects. The variable $\epsilon_{ij}$ is the normal error with zero means and the values are independent both within and between groups. The detailed procedure can be found in [19].

### 2.4. Principal Component Analysis

PCA is a machine learning technique for dimensionality reduction that converts a large dataset into a smaller one by transforming the primary features into new features, called principal components, which are combinations of the primary ones. The first few principal components typically retain most of the information from the larger dataset. PCA decomposes a multivariate dataset into a set of successive orthogonal components that explain the maximum amount of variance [20]. Using the singular value decomposition (SVD) of the data to project them to a lower-dimensional space, linear dimensionality reduction is performed. The input data are centered but not scaled for each feature before applying the SVD.

Given a dataset X, with *n* samples (observations or records) and *p* features (attributes), the *j*th principal component can be shown by the following:

(5) $$PCA_{j}=w_{j1}X_{1}+w_{j2}X_{2}+\cdots +w_{ji}X_{i}+\cdots +w_{jp}X_{p}$$

where $X_{i}$ is the *i*th feature of the dataset X and $w_{ji}$ is the weighting (loading) of the *j*th principal component of the *i*th feature. As shown in Equation (5), the principal components are a linear combination of the primary features. The loadings can be found using the eigenvectors of matrix Σ, where we have the following:

(6) $$\Sigma =X^{T}X$$

### 2.5. Self-Organizing Map

A self-organizing map (SOM) is a neural network unsupervised learning method. This method learns to classify input vectors according to how they are grouped in the input space. Neighboring neurons in the self-organizing map learn to recognize neighboring sections of the input space. Thus, SOM methods learn both the distribution and topology of the input vectors they are trained on. Distances between neurons (clusters or best-matching units (BMUs)) are calculated from their positions with a distance function. In this neural network, all neurons within a certain neighborhood of the winning neuron (BMU) are updated, using the Kohonen rule [21]. The weight vector ($\vec{w}$) of neuron (BMU) ij in the iteration (epoch) *t* is updated as follows:

(7) $${\vec{w}}_{ij}^{t}={\vec{w}}_{ij}^{t-1}+\eta^{t-1}f^{t-1}\left(\vec{x}-{\vec{w}}_{ij}^{t-1}\right)$$

where $\vec{x}$ is the input vector, η is the learning rate, and *f* is the neighborhood distance function. The details of the algorithm are found in [22].

## 3. Results and Discussion

### 3.1. Correlation Analysis

**Figure 2:**
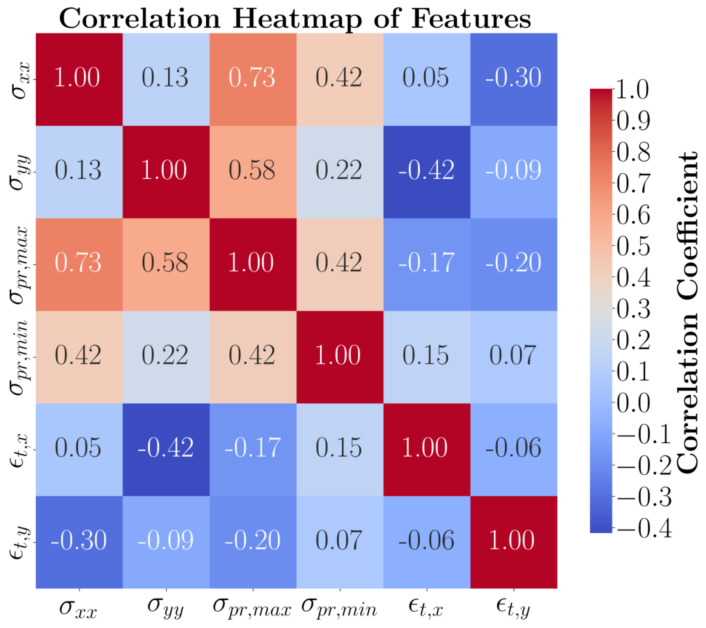
Heat map of the correlation between normal stresses, principal stresses, and total strains for the entire dataset (four geometries, each with three toolpaths).

A primary investigation into the interaction between geometry and toolpath on the residual mechanical properties of DED-built parts is based on the correlation between multiple variables, such as stresses and strains. Normal residual stresses, maximum and minimum principal stresses, and total directional strains are selected from the four geometries, each analyzed under three separate toolpath scenarios. The maximum and minimum residual stresses are considered as they play a crucial role in determining the impact of deposition direction on stresses [23]. The correlation heat map of the concatenated data for all the case studies (12 scenarios) is presented in Figure 2. Recall that the *x*-axis corresponds to the longest length of the shapes and *y* denotes the transverse direction. The maximum principal stress and the longitudinal residual stress ($\sigma_{xx}$) have the highest correlation, 0.73, implying the highest dependency of the optimum toolpath on the longitudinal direction.

**Figure 3:**
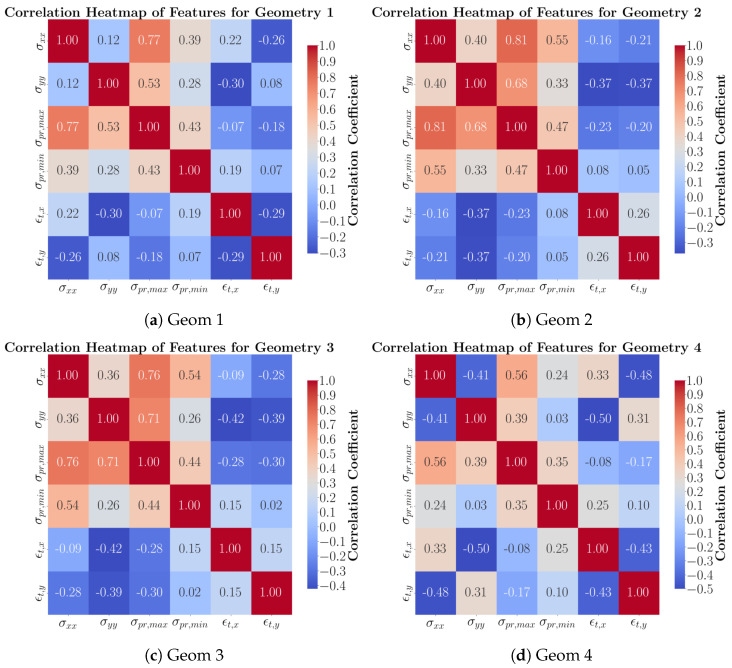
Heat map correlation normal stresses, principal stresses, and total strains for each geometry with three toolpaths.

The results of the entire dataset, including the geometries and the toolpaths, indicate that the longitudinal residual stress has a high correlation with maximum principal residual stress, which significantly impacts the optimum toolpath selection. Figure 3 displays the correlation between features for each geometry individually, incorporating all toolpaths for each geometry. The results show that the step-type geometries (Geom 2 and 3) demonstrate a high correlation between the principal and longitudinal and transverse stresses. It implies that a combination of deposition directions would result in the possible optimum toolpath selection for these geometries. The rectangle part with a big hollow in the middle with thin walls (Geom 4) represents the lowest correlation between the directional stresses and the maximum principal stresses.

**Figure 4:**
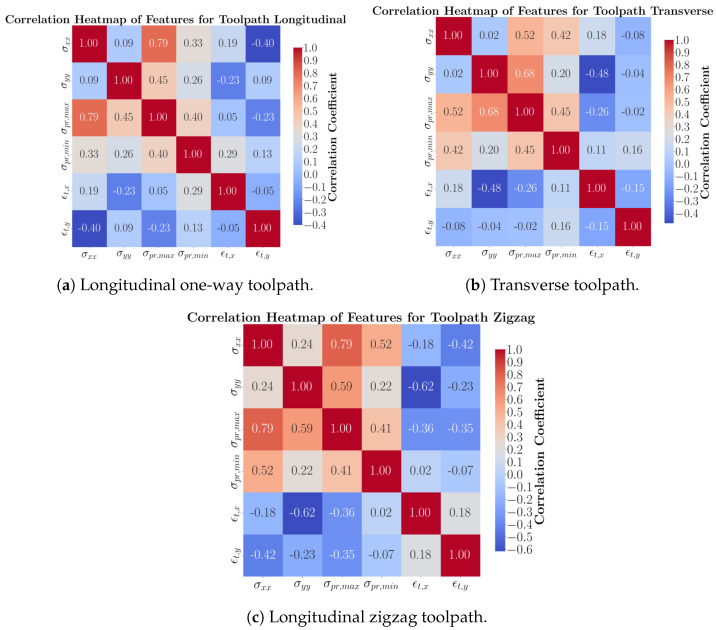
Heat map correlation normal stresses, principal stresses, and total strains for each toolpath with four geometries.

Similarly, the correlation between features for each toolpath individually, incorporating all geometries, is shown in Figure 4. The toolpath directional stresses are highly correlated with the maximum residual stress. For both the longitudinal one-way and longitudinal zigzag toolpaths, the *x*-axis residual stresses show a strong correlation with the maximum residual stress. In contrast, the transverse toolpath exhibits a high correlation between the transverse (*y*-axis) residual stress and the maximum principal stress.

The results reveal that—for thin parts—longitudinal depositions are the determining factor in forming residual stresses, whereas thick parts are affected by both longitudinal and transverse directions. Additionally, the results indicate that the deposition direction generates the highest residual stress in that specific direction.

### 3.2. Linear Mixed-Effects Model

The data reviewed so far indicate that the final mechanical properties of DED-manufactured parts depend on the toolpath, geometry type, and layer. To quantify the contributions of each factor (geometry type, toolpath pattern, and layer number) to the mechanical properties (e.g., residual stresses and strains), a linear mixed-effects model is used. This model considers the fixed effects (geometry, toolpath, and the DED-built layer number) on the random effects (distributed data on the parts). In this context, the random effect refers to the individual local nodes for all the case studies. For this purpose, the “*MixedLM*” module from the “statsmodels.formula.api” library [18] of the Python language was used to fit the model. The dependent variables are as follows: $\sigma_{xx}$, $\sigma_{yy}$, $\sigma_{pr,max}$, $\epsilon_{t,x}$, and $\epsilon_{t,y}$. All the data from the 12 case studies were concatenated into a unique dataset as two additional features of the toolpath pattern, and the geometry types were added to the observations of each data node from the FEM data. The fitting model results for each variable are presented in Table 2, Table 3, Table 4, Table 5 and Table 6. The intercept (reference) case is geometry 1 with the longitudinal toolpath. In comparison with the intercept case, the rest of the case studies are statistically analyzed.

The Coef. parameter in Table 2 for the intercept represents the estimated mean value of $\sigma_{xx}$ when all other variables are at their reference levels, with the remaining rows showing deviations from the intercept. Geometries 2 to 4 all show decreased levels of $\sigma_{xx}$; however, geometry 3 does not display significant change compared to the reference (Geom 1, Longitudinal) as the *p*-value (0.147) is above 0.05. All coefficients except geometry 3 are statistically significant at the 0.05 level, as indicated by P > |z| values less than 0.05. Only the zigzag pattern appears to have higher stress compared to the reference case. Since the group variance is low (0.003), minimum variation occurs between groups, indicating that most variability in $\sigma_{xx}$ is explained by fixed effects rather than random effects. The layer, zigzag pattern, and geometry 2 have the highest impacts on $\sigma_{xx}$, respectively.

Regarding variable $\sigma_{yy}$, as presented in Table 3, geometries 2 and 3 have the greatest impact, with increased levels of $\sigma_{yy}$ at 47.0 and 77.9 (MPa), respectively. The effects of the layer and toolpath are lower compared to the impact of geometry. The P-values of all fixed effects are below 0.05, indicating that these effects are significant. The larger group variance (1770.2) compared to the previous model ($\sigma_{xx}$) suggests more variability between groups in $\sigma_{yy}$ compared to $\sigma_{xx}$. It suggests that the random effects (local data distribution) are significant on $\sigma_{yy}$. A general comparison between the results of Table 2 and Table 3 highlights that geometry can have a positive effect on $\sigma_{xx}$ and a negative impact on $\sigma_{yy}$. The layer has a greater influence on $\sigma_{xx}$.

The results of Table 4 demonstrate that the geometry has the highest impact on $\sigma_{pr,max}$ compared to the toolpath, while the layer effect is predominant. The analyses of $\epsilon_{t,x}$ and $\epsilon_{t,y}$ in Table 5 and Table 6 show positive effects of geometries 2 to 4 on $\epsilon_{t,x}$ and negative effects on $\epsilon_{t,y}$ compared to the intercept (reference), displaying behavior similar to that described for the normal stresses.

The similar statistical linear mixed-effects analysis of the toolpath and geometry on the stresses and strains contrasts with the low correlation between the directional stresses and strains mentioned in the previous subsection. To gain a deeper understanding of whether stress or strain can represent the variability in the local data, principal component analysis (PCA) is utilized to identify the most important variables. PCA reduces the data features while introducing new features composed of linear sums of the primary features. The weighting factor of the primary features in the new features reveals which variables have the highest variance. The results of the PCA analysis are described in the next subsection.

### 3.3. Principal Component Analysis

The PCA class from the Python *scikit*-*learn* library [24] was used for the PCA machine learning analysis of the dataset of the current study. PCA was implemented to investigate which variables among five—$\sigma_{xx}$, $\sigma_{yy}$, $\sigma_{pr,max}$, $\epsilon_{t,x}$, and $\epsilon_{t,y}$—show the highest variance and are the most informative. Separate analyses were conducted for each geometry (including all toolpaths) and each toolpath (including all geometries).

**Figure 5:**
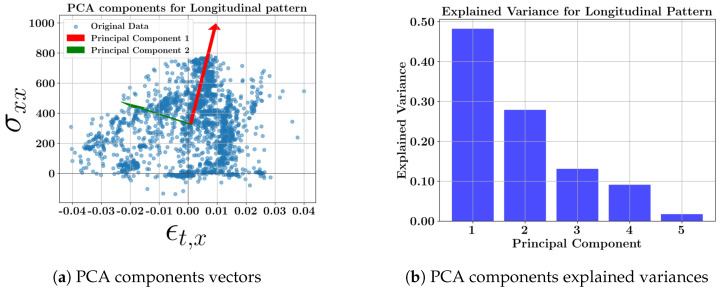
PCA results of the one-way longitudinal pattern at the middle layer; (**a**) scatter data showing two PCA component vectors; (**b**) variance of each new principal component.

The results for the concatenated data of all longitudinal pattern geometries were analyzed to gain an understanding of the “longitudinal scanning” pattern on the variability of the mechanical properties in DED-fabricated parts. Figure 5 shows the scatter plot of the data with two principal component vectors and the bar plot of the explained variance of the data at the middle layer (layer 3). The explained variance of each component indicates how informative the component is. The loading (weight) coefficients of the primary variables for each new principal component variable of the longitudinal toolpath are presented in Table 7. It should be noted that the reason the PCA component vectors are not exactly orthogonal in the 2D plot is that they are projected from a higher-dimensional space (6D in this case) onto a 2D plane.

The results indicate that the first principal component, which accounts for 48% of the information variance, is highly dependent on $\sigma_{xx}$, with a coefficient of 0.81. The second highest variability belongs to $\sigma_{yy}$, which has a loading of 0.80 in the second component. $\epsilon_{xx}$ and $\epsilon_{yy}$ contribute to the principal components with low impact as they mostly affect the third and fourth principal components. Similarly, the variance of $\sigma_{pr,max}$ has a lower contribution than the normal residual stresses. While the maximum principal stress ($\sigma_{pr,max}$) suggests an optimal approach for toolpath selection, the normal longitudinal stress ($\sigma_{xx}$) shows the highest variability at the observed nodes of the parts for the longitudinal pattern. In contrast, the variances for the strains ($\epsilon_{xx}$ and $\epsilon_{yy}$) are lower and negligible. Thus, longitudinal stress should be a matter of concern when investigating the longitudinal deposition for a longitudinal geometry.

**Figure 6:**
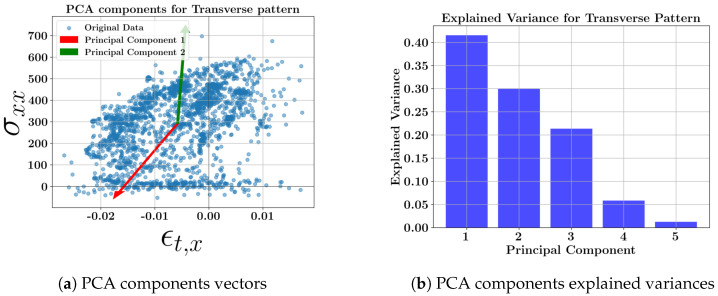
PCA results of the one-way longitudinal pattern at the middle layer; (**a**) scatter data showing two PCA component vectors; (**b**) variance of each new principal component.

Figure 6 shows the scatter plot of the data with two principal component vectors and the bar plot of the explained variance of the data at the middle layer (layer 3) for the transverse deposition pattern across all geometries. The loading (weight) coefficients of the primary variables for each new principal component variable of the transverse toolpath are presented in Table 8.

Regarding the data from the transverse deposition for all the geometries, the first principal component has the highest weighting of −0.62 for the longitudinal normal strain ($\epsilon_{t,x}$) followed by the second-highest weighting of 0.56 for the transverse directional residual stress $\sigma_{yy}$. Based on this evidence, the geometry shape type, specifically a longitudinal geometry, accounts for a higher directional strain variance than the deposition direction while the directional stress is mostly impacted by the deposition.

**Figure 7:**
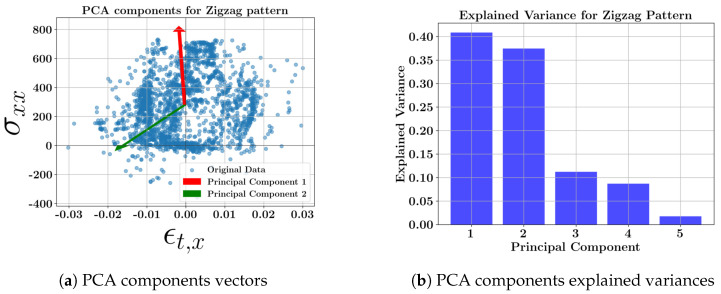
PCA results of the one-way longitudinal pattern at the middle layer; (**a**) scatter data showing two PCA component vectors; (**b**) variance of each new principal component.

Figure 7 shows the scatter plot of the data with two principal component vectors and the bar plot of the explained variance of the data at the middle layer (layer 3) for the longitudinal zigzag deposition pattern of all the geometries. The loading (weight) coefficients of the primary variables for each new principal component variable of the transverse toolpath are presented in Table 9.

The PCA results of the longitudinal zigzag show that the directional stress in the longitudinal axis obtains the highest variance and the strains have lower variances compared to the directional stresses. All the toolpath results demonstrate that the second-highest variance occurs in the orthogonal direction of the deposition. This means that longitudinal depositions result in the second highest variances of $\sigma_{yy}$, while transverse scanning results in the second highest variance of $\sigma_{xx}$, based on the explained variances of the principal components and the individual variable coefficients.

**Figure 8:**
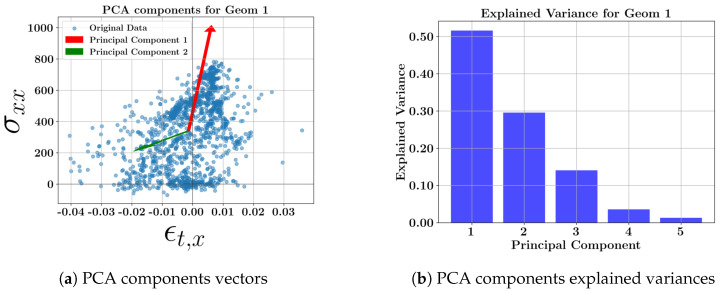
PCA component vectors and explained variance bars of geometry 1 at the middle layer.

**Figure 9:**
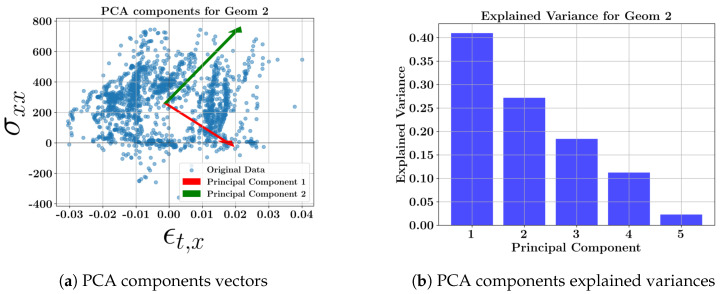
PCA component vectors and explained variance bars of geometry 2 at the middle layer.

**Figure 10:**
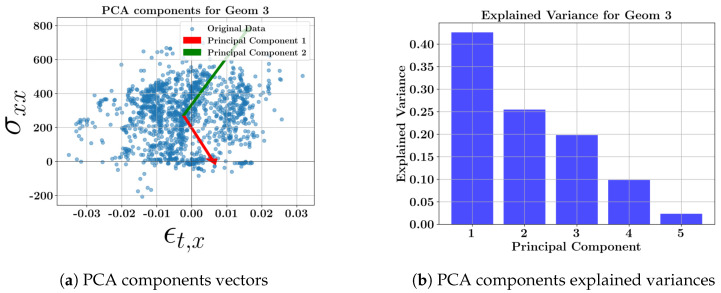
PCA component vectors and explained variance bars of geometry 3 at the middle layer.

**Figure 11:**
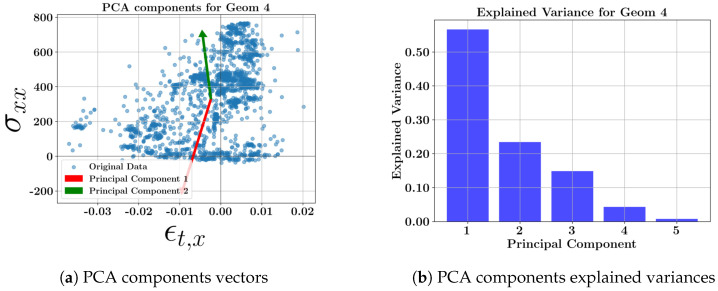
PCA component vectors and explained variance bars of geometry 4 at the middle layer.

The scatter plots for each geometry, including all toolpaths and the related explained variance bars, are presented in Figure 8, Figure 9, Figure 10 and Figure 11. The corresponding weighting values are presented in Table 10, Table 11, Table 12 and Table 13. The thin geometries (Geoms 1 and 4) show that the highest variance coefficient in the first principal belongs to $\sigma_{xx}$, while thick step walls show the highest coefficient pertaining to $\sigma_{yy}$. Similar to the toolpath results, the strains contribute to the principal components with low explained variance. It can be concluded that the residual stresses should be the focus due to their high variances at the middle layers of the DED parts, and the strains can be excluded from further multi-dimensional feature studies.

### 3.4. Self-Organizing Map Clustering

**Figure 12:**
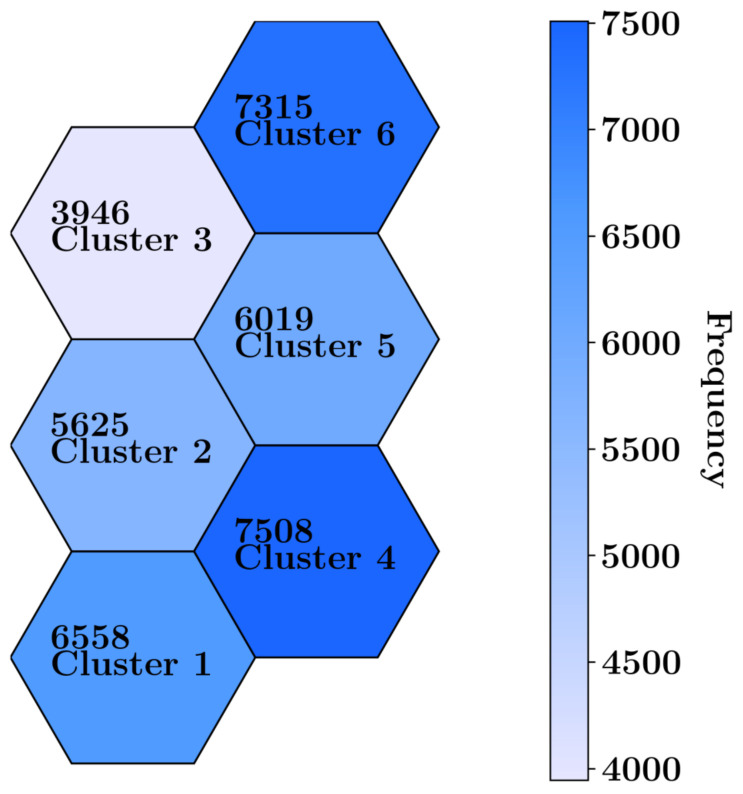
Structure of neurons (clusters) with the number of samples in each cluster for the local dataset.

The local points dataset from all 12 cases was evaluated using six clusters. The data were trained in Python using the MiniSom library [22], with a learning rate of 0.5 and 5000 iterations. The frequency of assigned observations (the FEM local nodes) to each cluster after training the SOM is shown in Figure 12. Clusters 4 and 6 contain the highest frequency of data, while cluster 3 has the fewest records (observations).

**Figure 13:**
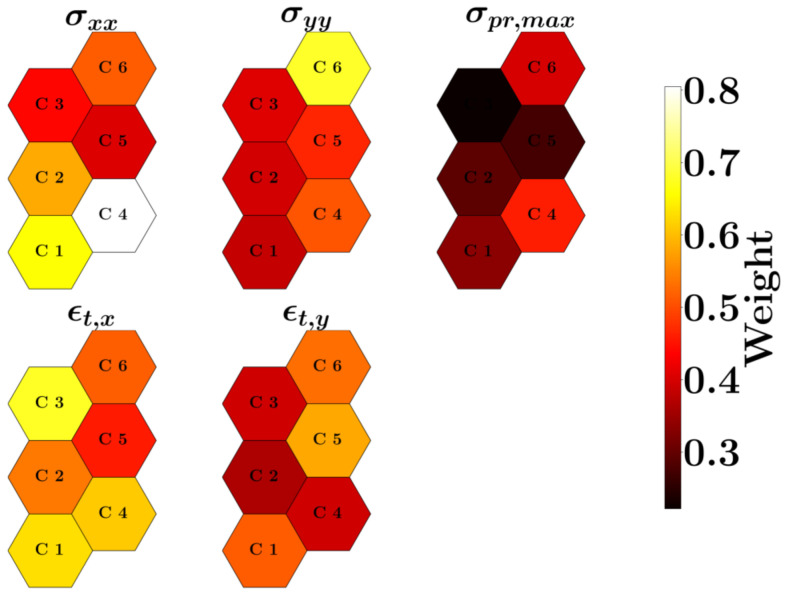
Weight of SOM neural network for each cluster (neuron)—C1 to C6 indicate cluster numbers from 1 to 6.

The weights of each feature in all the clusters (neurons) are presented in Figure 13. The $\sigma_{xx}$ stress has the highest weight in cluster 4 and $\sigma_{pr,max}$ has the lowest contribution in cluster 3. A visual consideration reveals that the weights of $\sigma_{pr,max}$ are lower compared to the rest of the features. Cluster 3 is mostly affected by $\epsilon_{t,x}$, and cluster 6 is highly impacted by $\sigma_{yy}$. Clusters 1 and 4 are predominantly determined by $\sigma_{xx}$ and $\epsilon_{t,x}$.

**Figure 14:**
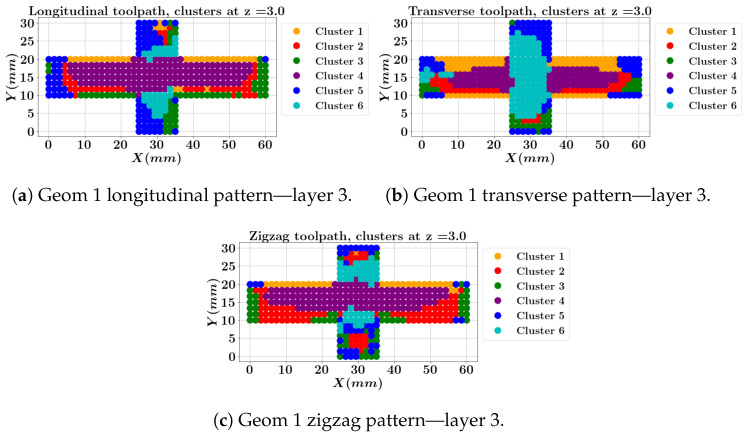
Local data clustering results for geometry 1 with three different toolpaths at layer 3.

**Figure 15:**
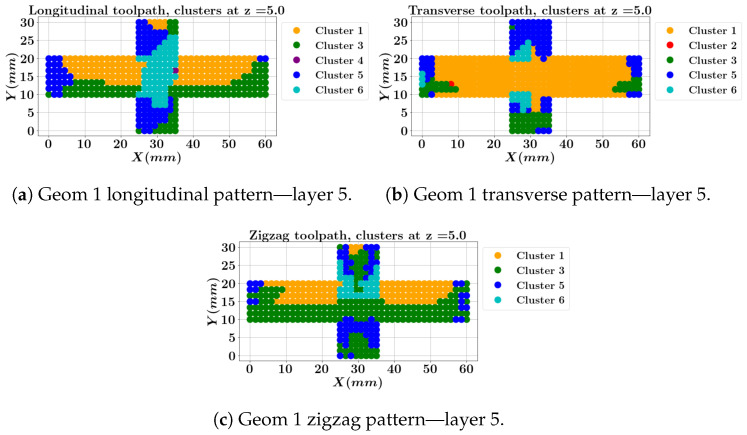
Local data clustering results for geometry 1 with three different toolpaths at layer 5 (top surface).

**Figure 16:**
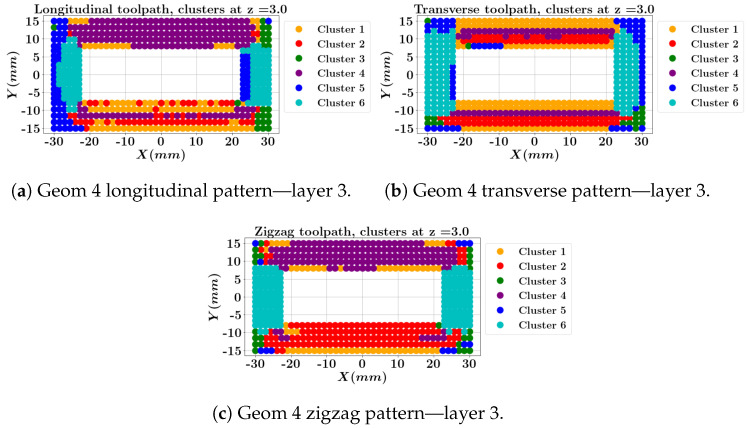
Local data clustering results for geometry 4 with three different toolpaths at layer 3.

**Figure 17:**
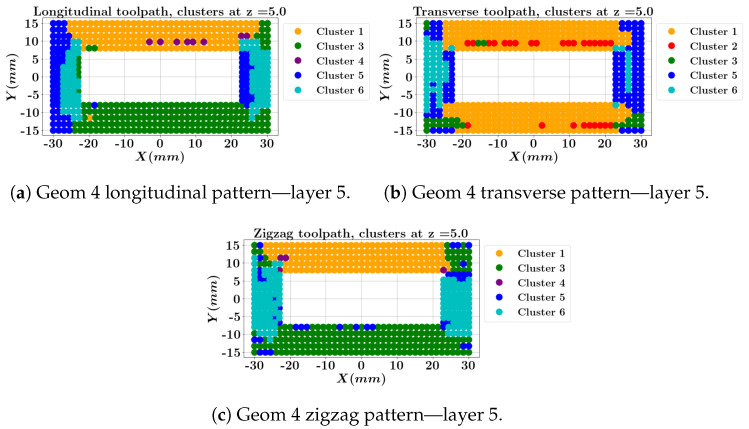
Local data clustering results for geometry 4 with three different toolpaths at layer 5 (top surface).

The features of the center of each cluster of the SOM results along with the defined mechanical status are presented in Table 14. In the contour plots (Figure 14, Figure 15, Figure 16 and Figure 17), the color represented in each row corresponds to the cluster’s color. Cluster 3 (green color) represents the low-stress and high-strain cluster, while Cluster 4 (purple color) represents the high-stress and low-strain cluster.

Based on the clustering analysis, the SOM model evaluated the nodes of the FEM results. Figure 14 illustrates the clusters each node is assigned to for geometry 1, encompassing three toolpath patterns at the middle layer (layer 3). It should be noted that the longitudinal one-way and zigzag depositions start at *X* = 25 mm and *Y* = 0 mm, while the transverse one-way deposition starts at *X* = 60 mm and *Y* = 20 mm. The transverse pattern at the third layer (Figure 14b) shows that the intersection zone within the cross-type part lies in a high directional $\sigma_{yy}$ stress with low strains cluster (cluster 6). The two longitudinal one-way and zigzag patterns (a, c) fall within the all-directional high stresses and high strains cluster (cluster 4) at the intersection. Both clusters at the intersection are characterized by high strains. The corners are assigned to cluster 5, which represents low stresses but high strains.

Figure 15 displays the clusters of each node for geometry 1 at the top surface (layer 5). The longitudinal zigzag pattern has the higher portion of cluster 3 (the green color) that represents the low-stress/low-strain category. The longitudinal one-way pattern exhibits the high transverse stress $\sigma_{yy}$ cluster assigned to the intersection area while the transverse pattern outcomes a converse result, indicating a high longitudinal stress cluster (cluster 1) at the intersection zone.

A comparison of Figure 14 and Figure 15 ascertains that the intersection might be optimized with a toolpath at the middle layer while worsening at the top layer with the same toolpath.

The cluster contours for the middle layer and the top layer of geometry 4 are depicted in Figure 16 and Figure 17. Note that the longitudinal one-way and zigzag depositions start at X=−30 (mm) and Y=−15 (mm) while the transverse one-way deposition starts at X=30 (mm) and Y=−15 (mm). Both one-way and zigzag longitudinal patterns at layer 3 (Figure 16a,c) show that the top long wall, where the deposition ends, is predominantly assigned to cluster 4 (the purple color, indicating high stresses and strains), which is less favorable compared to the transverse deposition outcome (Figure 16b). In contrast, the longitudinal patterns feature a low stress/strain cluster (the green color, Figure 17a,c) at the top layer within the first longitudinal wall where the deposition starts, while the transverse deposition shows cluster 1 for the longitudinal wall. However, the transverse deposition concludes in cluster 5 for the short transverse wall.

## 4. Conclusions

This research provides a comprehensive analysis of the impact of toolpath patterns, geometry types, and layering effects on the mechanical properties of parts fabricated through DED additive manufacturing using LME, PCA, and SOM clustering. The outcome shows the capability of the methods in quantifying and interpreting the contributions of these factors to residual stresses and strains.

The results indicate that all geometry types, toolpath patterns, and layer numbers significantly impact the distribution of mechanical properties. The toolpath had the highest impact on the longitudinal residual stress, $\sigma_{xx}$, while the shape type had the most substantial effect on the transverse residual stress, $\sigma_{yy}$. The LME model demonstrated that the layering effect is significant in all cases.

The PCA results further identified that the toolpath pattern has the greatest effect on directional stresses, while the geometry type accounts for the most significant impact on residual strains. It was also shown that residual stresses exhibit higher variance and are more informative than residual strains.

The SOM clustering offers additional insights into the local distribution of stresses and strains, highlighting regions within the parts where specific stress patterns are more likely to occur. The SOM results, based on the mechanical property similarity of the cloud nodes in the parts, suggest that while a toolpath may create a low stress/strain region at one layer, it could deteriorate the mechanical properties at another layer.

Overall, this study underscores the importance of selecting appropriate toolpaths and geometries in DED processes to enhance mechanical properties and reduce defects. The methodologies used demonstrate significant potential in establishing a robust framework for future studies aiming to optimize additive manufacturing processes through data-driven analysis. The conclusions are summarized in Table 15.

## Figures and Tables

**Table 1:** Process parameters used for the simulations.

Parameter	Data
Laser power	1000 W
Deposition speed	9.1 mm/s
Number of layers	5
Bead width	2 mm
Bead height	1 mm
Bead overlap	23%

**Table 2:** Mixed linear model regression results for variable $\sigma_{xx}$.

**Model:**	MixedLM		**Dependent Variable:**		Q($\sigma_{xx}$(MPa))	
**No. Observations:**	36,971		**Method:**		REML	
**No. Groups:**	10,206		**Scale:**		42,248.3905	
**Min. group size:**	1		**Log-Likelihood:**		−249,340.2347	
**Max. group size:**	4		**Converged:**		Yes	
**Mean group size:**	3.6					
	**Coef.**	**Std.Err.**	**z**	**P > |z|**	**[0.025**	**0.975]**
Intercept (“Geom 1,Longitudinal”)	394.452	3.257	121.115	0.000	388.069	400.836
C(Geometry,Treatment)[T.2]	−36.367	3.127	−11.610	0.000	−42.436	−30.297
C(Geometry, Treatment)[T.3]	−4.609	3.220	−1.450	0.147	−10.980	1.642
C(Geometry, Treatment)[T.4]	−9.010	3.113	−2.894	0.004	−15.111	−2.910
C(Toolpath, Treatment)[T.Trans]	−37.963	2.612	−14.537	0.000	−43.082	−32.845
C(Toolpath, Treatment)[T.Zigzag]	12.185	2.643	4.610	0.000	7.005	17.365
Q(“Layer”)	−38.673	0.658	−58.774	0.000	−39.962	−37.383
Group Var	0.003	0.940				

**Table 3:** Mixed linear model regression results for variable $\sigma_{yy}$.

**Model:**	MixedLM		**Dependent Variable:**		Q($\sigma_{yy}$(MPa))	
**No. Observations:**	36,971		**Method:**		REML	
**No. Groups:**	10,206		**Scale:**		31,461.3621	
**Min. group size:**	1		**Log-Likelihood:**		−244,834.7612	
**Max. group size:**	4		**Converged:**		Yes	
**Mean group size:**	3.6					
	**Coef.**	**Std.Err.**	**z**	**P > |z|**	**[0.025**	**0.975]**
Intercept (“Geom 1,Longitudinal”)	211.383	2.937	71.963	0.000	205.626	217.141
C(Geometry, Treatment)[T.2]	47.009	2.708	17.359	0.000	41.702	52.317
C(Geometry, Treatment)[T.3]	77.948	2.785	27.990	0.000	72.490	83.406
C(Geometry, Treatment)[T.4]	7.887	2.694	2.927	0.003	2.606	13.167
C(Toolpath, Treatment)[T.Trans]	−18.807	2.747	−7.861	0.000	−23.606	−14.008
C(Toolpath, Treatment)[T.Zigzag]	15.695	2.489	6.306	0.000	10.817	20.573
Q(“Layer”)	−21.713	0.582	−37.327	0.000	−22.853	−20.573
Group Var	1770.261	0.969				

**Table 4:** Mixed linear model regression results for variable $\sigma_{pr,max}$.

**Model:**	MixedLM		**Dependent Variable:**		Q($\sigma_{pr,max}$(MPa))	
**No. Observations:**	36,971		**Method:**		REML	
**No. Groups:**	10,206		**Scale:**		27,322.7092	
**Min. group size:**	1		**Log-Likelihood:**		−241,291.6528	
**Max. group size:**	4		**Converged:**		Yes	
**Mean group size:**	3.6					
	**Coef.**	**Std.Err.**	**z**	**P > |z|**	**[0.025**	**0.975]**
Intercept (“Geom 1,Longitudinal”)	504.901	2.619	192.757	0.000	499.767	510.034
C(Geometry, Treatment)[T.2]	−33.269	2.515	−13.228	0.000	−38.199	−28.340
C(Geometry, Treatment)[T.3]	9.448	2.589	3.649	0.000	4.373	14.524
C(Geometry, Treatment)[T.4]	6.651	2.503	2.657	0.008	1.744	11.557
C(Toolpath, Treatment)[T.Trans]	−16.531	2.486	−7.870	0.000	−20.648	−12.414
C(Toolpath, Treatment)[T.Zigzag]	13.096	2.128	6.155	0.000	8.926	17.266
Q(“Layer”)	−34.261	0.529	−64.738	0.000	−35.298	−33.224
Group Var	0.000	0.734				

**Table 5:** Mixed linear model regression results for variable $\epsilon_{t,x}$.

**Model:**	MixedLM		**Dependent Variable:**		Q($\epsilon_{t,x}$)	
**No. Observations:**	36,971		**Method:**		REML	
**No. Groups:**	10,206		**Scale:**		0.0001	
**Min. group size:**	1		**Log-Likelihood:**		118,013.0819	
**Max. group size:**	4		**Converged:**		Yes	
**Mean group size:**	3.6					
	**Coef.**	**Std.Err.**	**z**	**P > |z|**	**[0.025**	**0.975]**
Intercept (“Geom 1,Longitudinal”)	−0.006	0.000	−34.307	0.000	−0.006	−0.005
C(Geometry, Treatment)[T.2]	−0.003	0.000	−23.181	0.000	−0.004	−0.003
C(Geometry, Treatment)[T.3]	−0.003	0.000	−16.873	0.000	−0.003	−0.002
C(Geometry, Treatment)[T.4]	−0.001	0.000	−6.942	0.000	−0.001	−0.001
C(Toolpath, Treatment)[T.Trans]	−0.006	0.000	−41.419	0.000	−0.006	−0.005
C(Toolpath, Treatment)[T.Zigzag]	−0.002	0.000	−15.916	0.000	−0.002	−0.002
Q(“Layer”)	0.003	0.000	86.830	0.000	0.003	0.003
Group Var	0.000	0.000				

**Table 6:** Mixed linear model regression results for variable $\epsilon_{t,y}$.

**Model:**	MixedLM		**Dependent Variable:**		Q($\epsilon_{t,y}$)	
**No. Observations:**	36,971		**Method:**		REML	
**No. Groups:**	10,206		**Scale:**		0.0001	
**Min. group size:**	1		**Log-Likelihood:**		121,398.0879	
**Max. group size:**	4		**Converged:**		Yes	
**Mean group size:**	3.6					
	**Coef.**	**Std.Err.**	**z**	**P > |z|**	**[0.025**	**0.975]**
Intercept	−0.017	0.000	−121.048	0.000	−0.018	−0.017
C(Geometry, Treatment)[T.2]	0.003	0.000	23.698	0.000	0.003	0.004
C(Geometry, Treatment)[T.3]	0.005	0.000	33.144	0.000	0.004	0.005
C(Geometry, Treatment)[T.4]	0.003	0.000	21.035	0.000	0.003	0.003
C(Toolpath, Treatment)[T.Trans]	0.006	0.000	50.564	0.000	0.006	0.006
C(Toolpath, Treatment)[T.Zigzag]	−0.003	0.000	−27.517	0.000	−0.003	−0.003
Q(“Layer”)	0.002	0.000	74.192	0.000	0.002	0.002
Group Var	0.000	0.000				

**Table 7:** Loadings (weights) for each principal component—longitudinal toolpath pattern data.

	PCA 1	PCA 2	PCA 3	PCA 4	PCA 5
$\sigma_{xx}$	0.81	0.14	0.26	0.23	−0.45
$\sigma_{yy}$	−0.09	0.80	−0.51	−0.13	−0.28
$\sigma_{pr,max}$	0.41	0.31	−0.07	0.12	0.85
$\epsilon_{t,x}$	0.20	−0.44	−0.79	0.34	−0.06
$\epsilon_{t,y}$	−0.36	0.21	0.17	0.89	−0.01

**Table 8:** Loadings (weights) for each principal component—transverse toolpath pattern data.

	PCA 1	PCA 2	PCA 3	PCA 4	PCA 5
$\sigma_{xx}$	−0.45	0.61	−0.51	−0.30	0.27
$\sigma_{yy}$	0.56	0.56	0.30	0.22	0.49
$\sigma_{pr,max}$	0.16	0.50	−0.13	0.26	−0.80
$\epsilon_{t,x}$	−0.62	0.08	0.28	0.72	0.12
$\epsilon_{t,y}$	0.27	−0.25	−0.74	0.53	0.19

**Table 9:** Loadings (weights) for each principal component—Zigzag toolpath pattern data.

	PCA 1	PCA 2	PCA 3	PCA 4	PCA 5
$\sigma_{xx}$	0.69	−0.38	−0.16	0.27	−0.53
$\sigma_{yy}$	0.27	0.76	0.35	−0.24	−0.42
$\sigma_{pr,max}$	0.63	0.08	0.26	0.06	0.72
$\epsilon_{t,x}$	−0.05	−0.53	0.71	−0.45	−0.12
$\epsilon_{t,y}$	−0.22	0.05	0.52	0.82	−0.07

**Table 10:** Loadings (weights) for each principal component—-Geom 1 data.

	PCA 1	PCA 2	PCA 3	PCA 4	PCA 5
$\sigma_{xx}$	0.81	−0.12	−0.35	0.18	−0.41
$\sigma_{yy}$	0.21	0.77	0.52	−0.08	−0.28
$\sigma_{pr,max}$	0.46	0.21	−0.05	−0.10	0.86
$\epsilon_{t,x}$	0.20	−0.35	0.26	0.87	−0.11
$\epsilon_{t,y}$	−0.20	0.47	−0.73	−0.44	−0.09

**Table 11:** Loadings (weights) for each principal component—Geom 2 data.

	PCA 1	PCA 2	PCA 3	PCA 4	PCA 5
$\sigma_{xx}$	−0.33	0.63	0.08	−0.41	−0.57
$\sigma_{yy}$	−0.61	−0.29	−0.29	0.55	−0.40
$\sigma_{pr,max}$	−0.55	0.43	−0.01	0.13	0.70
$\epsilon_{t,x}$	0.45	0.52	−0.59	0.41	−0.06
$\epsilon_{t,y}$	0.14	0.25	0.75	0.59	−0.13

**Table 12:** Loadings (weights) for each principal component—Geom 3 data.

	PCA 1	PCA 2	PCA 3	PCA 4	PCA 5
$\sigma_{xx}$	−0.36	0.70	0.20	−0.37	0.45
$\sigma_{yy}$	−0.64	−0.39	−0.21	0.39	0.50
$\sigma_{pr,max}$	−0.63	0.21	0.06	0.16	−0.73
$\epsilon_{t,x}$	0.25	0.56	−0.36	0.70	0.06
$\epsilon_{t,y}$	0.07	−0.04	0.88	0.45	0.10

**Table 13:** Loadings (weights) for each principal component—Geom 4 data.

	PCA 1	PCA 2	PCA 3	PCA 4	PCA 5
$\sigma_{xx}$	−0.67	0.47	−0.36	0.13	0.44
$\sigma_{yy}$	0.55	0.61	0.33	0.02	0.46
$\sigma_{pr,max}$	−0.06	0.63	−0.05	−0.28	−0.72
$\epsilon_{t,x}$	−0.33	−0.10	0.48	0.78	0.21
$\epsilon_{t,y}$	0.36	−0.06	−0.73	−0.55	0.19

**Table 14:** SOM cluster centers—the rows’ colors align with the contours plotted in the results.

Cluster	Center σ_*xx*_	Center σ_*yy*_	Center σ_*pr,max*_	Center ϵ_*t,x*_ ($\times 10^{3}$)	Center ϵ_*t,y*_ ($\times 10^{3}$)	Status
1	385.29	49.29	421.18	5.18	−4.74	High $\sigma_{xx}$—Low Strain
2	280.36	66.24	355.53	−2.98	−18.75	High $\sigma_{xx}$—High Strain
3	80.19	79.80	245.09	9.89	−15.97	Low Stresses—High Strain
4	591.04	211.21	614.08	3.59	−15.90	High Stresses—High Strain
5	34.97	158.20	325.78	−10.61	1.25	Low $\sigma_{xx}$—High Strain
6	186.04	436.02	521.46	−5.26	−3.39	High $\sigma_{yy}$—Low Strain

The color of each row demonstrate the cluster color, later shown on the results.

**Table 15:** Summary of key insights and suggestions.

Factor	Effect on Properties	Suggestions
**Toolpath Pattern**	Highest impact on longitudinal residual stress ($\sigma_{xx}$)	Use longitudinal toolpaths to minimize longitudinal stresses, especially for thin geometries.
**Geometry Type**	Highest impact on transverse residual stress ($\sigma_{yy}$)	Step-type geometries need mixed toolpaths for balancing stress distribution.
**Layering Effect**	Significant across all mechanical properties	Control layering thickness to minimize internal stress buildup, especially in thick parts.

## Data Availability

The raw data supporting the conclusions of this article will be made available by the authors on request.

## Abbreviations

The following abbreviations are used in this manuscript:

AM	additive manufacturing
ANN	artificial neural network
CAD	computer-aided design
CNN	convolutional neural network
CPU	central processing unit
DED	direct energy deposition
ELM	linear mixed-effects model
FEM	finite element method
PCA	principal component analysis
RAM	random-access memory
SOM	self-organizing map
